# 102. A Retrospective Case Series of West Nile Neuroinvasive Disease in Two Tertiary Health Centers in Miami

**DOI:** 10.1093/ofid/ofab466.102

**Published:** 2021-12-04

**Authors:** Leopoldo Cordova, Aliya F Rehman, Shuba Balan, Folusakin Ayoade, Lizy Paniagua, Julia Bini, Cynthia Rivera

**Affiliations:** 1 University of miami / jackson memorial Hospital, Miami, FL; 2 Mount Sinai Medical Center, Miami, Florida; 3 Department of Infectious Diseases, University of Miami, Miami, Florida; 4 University of Miami, Miami, Florida; 5 MSMC, Miami, Florida

## Abstract

**Background:**

According to the Centers for Disease Control and Prevention, Florida was the third leading state in reported West Nile Neuroinvasive Disease (WNND) infections in 2020. WNND accounts for less than 1% of all West Nile virus (WNV) infections but carries a 10% mortality rate. The clinical characteristics of WNND have not been well described in Florida, an area with high mosquito activity. We hereby describe the clinical characteristics of WNND at two large hospitals in Miami.

**Methods:**

A 10-year retrospective study was performed at the University of Miami Hospital and Mount Sinai Medical Center to identify adult patients with confirmed WNV infection and neuroinvasion. Patient demographics, symptoms, neurological exam findings, laboratory diagnostics, intensive care unit (ICU), and hospital length of stay (LOS), and outcomes were described.

**Results:**

Eleven patients (73% male, mean age 64.4 ± 16.3 years) were identified between January 2010 to December 2020. The most prevalent comorbidities were HTN (64%) and DM (27%). The most common positive findings on the review of symptoms were fever (100%), confusion (81.8%), and headache (63.6%). The mean hospital LOS was 15.5 ±11.3 days, while the mean ICU LOS was 7.2 ± 11.9 days. The majority of patients (75%) spent more than 2 weeks in the ICU. Subject age was correlated with hospital LOS with a Pearson correlation of 0.624 (p=0.04). The survival rate was 91%. At the time of discharge, 80% of patients continued to have neurological symptoms.

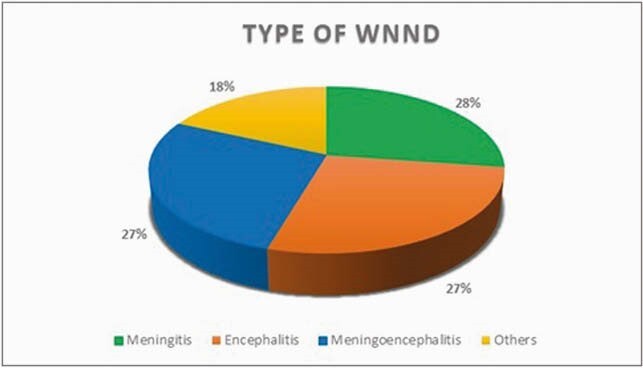

Figure 1: The percentage of subjects with different types of WNND. The section titled others, includes atypical presentations such as amnesia, focal neurological deficits (ataxia, hemiparesis), and myelopathy.

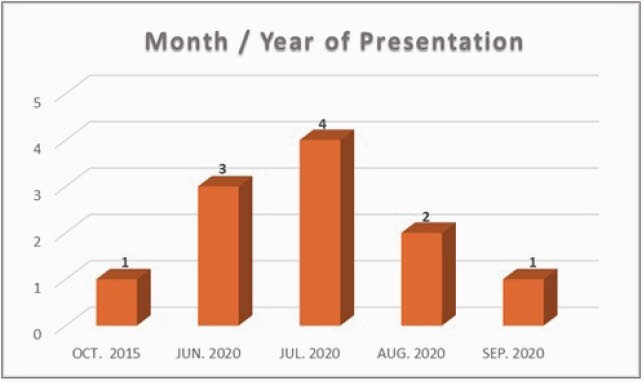

Figure 2: Month and year of presentation at the time of hospital admission.

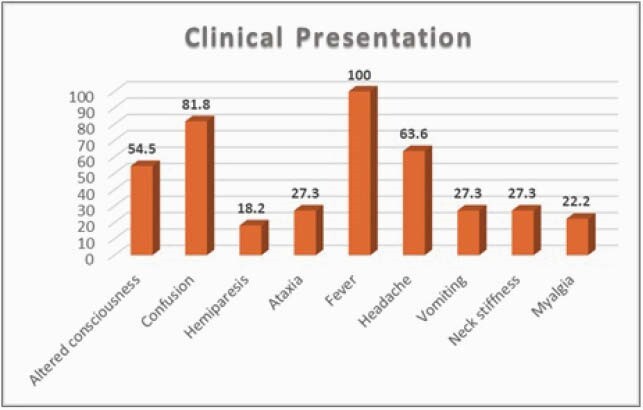

Figure 3: Clinical presentation (%).

**Conclusion:**

This is the largest case series of WNND in Florida. Most cases occurred during summer 2020, which corresponds to the peak of the COVID-19 pandemic. Despite pandemic restrictions, we may have seen an increase in WNV cases due to higher-than-normal temperatures promoting mosquito abundance, increased outdoor activities due to the COVID-19 pandemic, and/or the redistribution of public health resources towards the pandemic rather than mosquito control. Residual neurological symptoms and impaired functional outcomes are common. Within the limitation of our small sample size, subject age appeared to correlate with hospital LOS. This correlation should be further explored in a larger case series. A high index of suspicion for WNND is suggested for patients presenting with fever and neurologic symptoms in Florida.

**Disclosures:**

**Cynthia Rivera, MD**, **Gilead Sciences** (Advisor or Review Panel member)**Viiv Healthcare** (Advisor or Review Panel member)

